# Macular pucker and cataract treated with phacoemulsification and IOL implantation combined with small-gauge pars plana vitrectomy: a comparison of outcomes with and without femtosecond laser assistance

**DOI:** 10.3389/fmed.2025.1497776

**Published:** 2025-05-01

**Authors:** Howard Wen-Haur Chao, Cheng-Kuo Cheng, Shiow-Wen Liou, Hsiao-Ming Chao

**Affiliations:** ^1^Department of Medicine, School of Medicine, Aston University, Birmingham, United Kingdom; ^2^Department of Medical Education, Leeds University, Leeds, United Kingdom; ^3^Department of Ophthalmology, Shin Kong Wu Ho-Su Memorial Hospital, Taipei, Taiwan; ^4^School of Medicine, Catholic Fu-Jen University, Taipei, Taiwan; ^5^School of Medicine, National Taiwan University, Taipei, Taiwan; ^6^Department of Chinese Medicine, School of Chinese Medicine, China Medical University, Taichung, Taiwan; ^7^Institute of Pharmacology, School of Medicine, National Yang Ming Chiao Tung University, Taipei, Taiwan

**Keywords:** femtosecond laser, phacoemulsification, IOL implant, small gauge pars plana vitrectomy, oriental, macular pucker

## Abstract

**Objectives:**

Age-related cataracts and macular pucker are increasingly common. Standard treatment combines phacoemulsification, IOL implantation and small gauge vitrectomy. Recent advancements and acceptance of femtosecond laser (FSL) assistance in cataract surgery have improved precision and outcomes. However, evidence regarding the efficacy and safety of FSL-assisted phacovitrectomy, particularly in Oriental patient populations with distinct anatomical and genetic characteristics, remain limited. This study aims to address this critical gap by comparing the safety and post-operative outcomes of 23- or 25-gauge phacovitrectomy for stage 3 macular pucker and medium density cataract with versus without FSL-assistance (FSLA), in an Oriental patient cohort.

**Methods:**

Patients with stage 3 macular pucker and medium-density cataract were recruited and divided into two age-matched groups: group 1 (*n* = 13) underwent conventional phacovitrectomy without FSLA, and Group 2 (*n* = 13) underwent phacovitrectomy with FSLA. Evaluations included pre- and postoperative best-corrected visual acuity (BCVA; Snellen E and LogMAR), cataract surgical time, phacoemulsification energy cost, corneal wavefront data, endothelial cell density (ECD), and surgical complications.

**Results:**

Significant improvements in postoperative visual acuity were observed in both groups (*P* < 0.05), with Group 2 (FSLA) demonstrating superior outcomes (0.48 ± 0.05/−0.45 ± 0.06; Snellen E/logMAR) compared to Group 1 (0.26 ± 0.07/−0.66 ± 0.15; Snellen E/logMAR). FSLA significantly reduced surgical duration (429.46 s vs. 740.00 s) and cumulative dissipated energy (CDE; 18.90 ± 1.59 vs. 25.24 ± 1.42) without significantly altering higher-order aberrations (0.24 to 0.22 μm). Although ECD decreased postoperatively in both groups, FSLA phacovitrectomy resulted in significantly less endothelial cell loss (227.77 ± 46.85 cells/mm^2^) compared to conventional phacovitrectomy (389.15 ± 47.87 cells/mm^2^). No serious complications were reported in either group.

**Conclusion:**

FSLA phacovitrectomy presents a safe and more efficient alternative over traditional procedures for Oriental patients with medium density nuclear cataract patients with stage 3 macular pucker. Through enhanced IOL centration, shortened surgical times and decreased ECD loss, FSLA led to superior postoperative visual outcomes compared to traditional phacovitrectomy. This study addresses a critical gap in the literature by providing evidence for the benefits of FSLA in Oriental populations, offering valuable insights into its applicability in patients with distinct anatomical variations.

## 1 Introduction

With the advancement of diagnostic techniques and an aging population, the prevalence of conditions affecting elderly demographics is on the rise. Among these conditions, age-related cataract and macular pucker are becoming increasingly recognized. It is increasingly common to encounter patients presenting with both macular pucker and concurrent cataracts, and these patients necessitate integrated management strategies to address the co-existing conditions effectively. Macular pucker is characterized by the formation of fibrotic tissue (epiretinal membrane; ERM) above the internal limiting membrane (ILM) of the macula, while cataract is characterized by the opacification of the crystalline lens. Collectively, these conditions contribute to substantial vision impairment and functional disability in aging individuals.

The management of concurrent macular pucker and cataract necessitates surgical int ervention and typically involves vitrectomy combined with phacoemulsification. Vitrectomy using small gauge instrument is currently recommended as the standard procedural approach due to its numerous benefits, namely, reduction in surgery duration, lowered post-surgical infection rates, and reduced discomfort compared to traditional 20-G vitrectomy (1). Phacoemulsification remains the standard treatment for cataracts. Given that vitrectomy often accelerates cataract development in most patients, it is advantageous to simultaneously perform phacoemulsification and IOL implantation during the same surgery as it obviates the need for separate surgeries. This approach is presumed to facilitate significantly safer vitreoretinal operations and expedite visual recovery for patients. It also enhances maneuverability within the vitreous cavity during pars plana vitrectomy (PPVT) combined with ERM/ILM peeling. When phacoemulsification is combined with small gauge vitrectomy—known as phacovitrectomy—it constitutes a safe and efficient combined operation for addressing vitreoretinopathies related to cataracts.

While conventional phacovitrectomy has become a standard approach, recent technological advancements in femtosecond laser (FSL) technology have expanded its application beyond cataract surgery to vitreoretinal procedures. Femtosecond laser (FSL)-assisted cataract surgery (FSLACS) has gained acceptance due to its precision in capsulorhexis creation, nuclear fragmentation and corneal incisions (2). By ensuring a well-centered and consistently sized capsulotomy, FSLACS helps maintain optimal IOL positioning, which is crucial for long-term visual outcomes. Additionally, FSL reduces phacoemulsification ultrasound energy by presegmenting the crystalline lens, minimizing endothelial cell damage and corneal edema, facilitating faster visual recovery. Additionally, accurately performed main and side port corneal wounds, as well as corneal arcuate incisions at the intended locations and depths/lengths, effectively control postoperative astigmatism. FSL assistance (FSLA) enhances the safety and postoperative outcomes of phacoemulsification combined with intraocular lens implantation (PhacoIOL) In the context of vitrectomy, studies have demonstrated that FSLA provides a safe alternative to conventional phacovitrectomy in vitrectomized eyes (3–8). More specifically studies have indicated that FSLA reduces intraoperative ultrasound energy, limit endothelial cell loss and improve intraocular (IOL) stability in patients (3–8).

Despite demonstrated benefits, evidence on the efficacy and safety of FSL-assisted phacovitrectomy in East Asian patients remains limited. Existing studies have predominantly focused on Caucasian or South Asian populations, with limited data available for East Asian (oriental) patients (3–8), who often exhibit distinct anatomical and genetic characteristics (2–4, 6, 7). These include smaller visible eyes with narrower vertical and horizontal palpebral fissures (9), genetically smaller anterior chambers (10), and harder nuclei due to a delayed efficient management with current adopted technology (11, 12). Such differences may increase surgical complexity and the risk of complications (13, 14), underscoring the need for tailored approaches to improve outcomes (13, 14).

To the best of our knowledge, our study is the first to investigate and compare conventional phacovitrectomy versus FSL-assisted combined phacovitrectomy in Oriental populations. Only three studies published to date have compared conventional phacovitrectomy versus FSL-assistance in cases requiring phacoemulsification and vitrectomy (7, 8, 15). However, these studies focused on vitrectomized eyes, and only Cai et al. (15) investigated Chinese patients (7, 8, 15). The scarcity of data on the benefits and limitations of FSL-assisted phacovitrectomy in this population highlights a critical gap in the literature, particularly for patients with stage 3 macular pucker and medium-density nuclear cataract. Further research is needed to better understand whether FSL-assisted phacovitrectomy can address these unique challenges and improve surgical outcomes in Oriental patients (3, 4, 6, 9–11, 16).

To address this gap, our present study aims to investigate the surgical outcomes of Oriental patients with stage 3 macular pucker, as classified by Govetto et al. (16), and medium-density nuclear cataract undergoing combined phacovitrectomy plus peeling. This study aims to compare the effectiveness of phacoemulsification plus IOL implantation combined with vitrectomy and ILM peeling, both with and without femtosecond laser assistance. Key parameters were evaluated, namely, best corrected visual acuity (BCVA) in Snellen E and logarithm of the minimum angle of resolution (LogMAR), total cataract surgical time, higher order aberrations (HOAs), cumulative dissipated energy (CDE) and corneal endothelial cell density (ECD). By evaluating these parameters, this study aims to determine whether FSL-assisted phacovitrectomy improves visual outcomes, reduces surgical trauma and safety in Oriental patients with concurrent macular pucker and cataract.

## 2 Materials and methods

### 2.1 Inclusion and exclusion criteria of this study

A retrospective chart review of candidates with vitreoretinal pathology was carried out. Permission for this study was obtained from the Institutional Review Board at Shin Kong Wu Huo-Shih Memorial Hospital (20210504R). In addition, confidentiality agreements were respectively signed by the Principal Investigator and Co-Principal Investigator. The scheduled project was conducted from June 1st, 2021, to May 31st, 2022. The follow-up period for patients was on average 34.8 ± 3.4 months.

**Figure 1:**
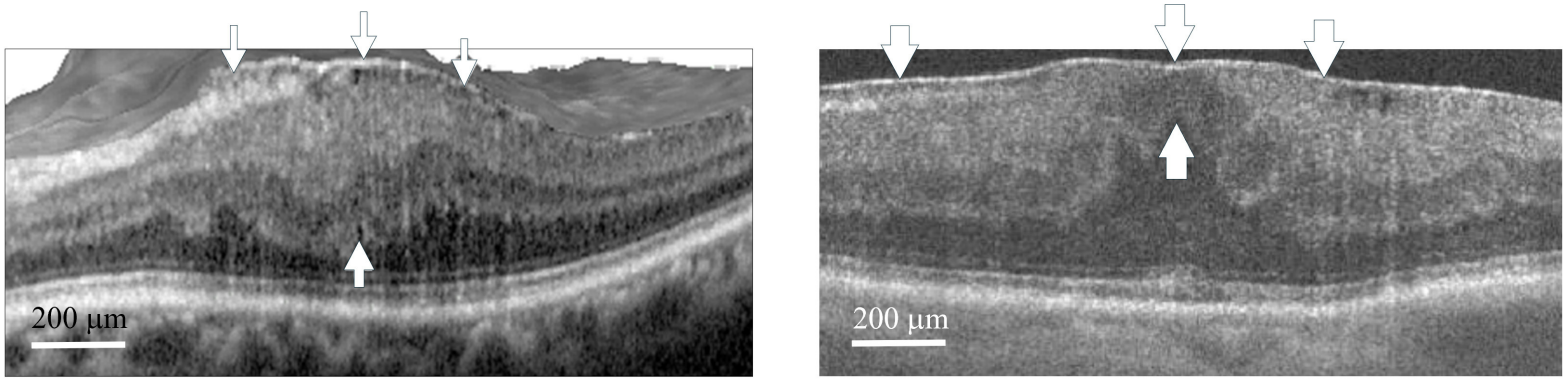
Optical coherence tomography scan of macular pucker. The left panel (Group 1) shows a representative case (Case 2) with stage 3 macular pucker and a central retinal thickness (CRT) of 527 μm, who later underwent phacovitrectomy with ERM/ILM peeling. The right panel (Group 2) shows a representative case (Case 3) with stage 3 macular pucker and a CRT of 520 μm, who later underwent femtosecond laser assisted phacovitrectomy with ERM/ILM peeling. The ectopic inner foveal layers (thin arrows for Case 2, thick arrows for Case 3) extend from the superior border of the outer nuclear layer to the ILM, with the upper arrows pointing to the ILM and the lower arrows indicating the superior border of the outer nuclear layer. CRT was measured from the ILM to the retinal pigment epithelium at the center of the fovea. Arrowheads at the top indicate the ERMs in both cases. ERM, epiretinal membrane; ILM, internal limiting membrane. Scale bar 200 μm.

The investigation was performed to assess the safety and the postoperative outcomes of 23- or 25-gauge PhacoIOL-PPVT-peeling in patients of stage 3 macular pucker (Figure 1) and medium density nuclear cataract (nuclear cataract scale 3–4 by Lens Opacities Classification System III) with versus without FSLA. A thorough evaluation was done via analyzing preoperative features, operative indications, postoperative data, and any associated side effects. The inclusion criterion for candidates was medium density nuclear cataracts combined with macular pucker. By selecting stage 3 ERM cases with comparable pre-operative puckering staging/severity in both study groups, the approach facilitated a more robust assessment of the impact of FSLA in phacovitrectomy.

### 2.2 Pre-operative and post-operative comprehensive ophthalmologic examinations

The pre-operative and post-operative outcomes were evaluated using various examinations, including BCVA in Snellen E and LogMAR formats, total cataract surgical time and HOAs. In addition, complications such as suction cup loss, disrupted anterior capsule CCC, anterior capsule tears extending to posterior capsule rupture, and nucleus fragment drops were investigated. The FSLA cataract surgical time was defined as the period from performing CCC, lens fragmentation, and opening main/side ports to the completion of phacoemulsification. In contrast, traditional phacoemulsification surgical time was measured starting from the opening of the main/side ports (by 2.2/1 mm knife) and performing CCC (by CCC forceps) to the completion of phacoemulsification (including sculpting/chopping, nucleus quad/fragment removal & cortex aspiration till a transparent posterior capsule is achieved). The total cataract surgical time was compared between two defined groups. As for the exclusion criteria, patients that had lost follow-up for 3 months were omitted. The preoperative conditions include a standardized CCC diameter at 5 mm, a safety zone at 550 μm, and a medium density nuclear cataract nucleus cut into 8 halves combined with either a circumferential ring (Z8, Ziemer, Switzerland; Catalys, Johnson & Johnson, New Jersey) or grid (LenSAR Inc., Orlando)-shaped cut.HOAs calculated by total root mean square (RMS) were also compared before and after femtosecond laser-assisted phacovitrectomy (FsPhacoVT). Monofocal aspheric IOL (ZCB00, J & J) was implanted in the present investigation.

The same surgeon (HM) performed three transconjunctival ports 3.5 mm from the limbus (Alcon Laboratories). Vitrectomy was performed using the Constellation vitrectomy system (Alcon Laboratories) and Zeiss microscope (OPMI LUMERA, Göschwitzer Straße 51–52 07745 Jena, Germany) with the help of wide angle contact lens (Mini Quad^®^ XL Lens; B106134, VOLK VITHAN-MQXL, USA) viewing fundus.

The exclusion criterion included candidates with other vitreoretinal disorders such as macular hole, retinal detachment, and vitreous hemorrhage. Additionally, patients of macular pucker stages 1, 2 and 4 were excluded. Patients at stages 1 and 2 generally had not experienced significant visual distortion or impaired vision, therefore observation was recommended for them. The patients at stage 4 exhibited disruptions across retinal layers and experienced the most severe vision impairment, making complete recovery extremely challenging. Thus, only stage 3 macular pucker was presently analyzed in this study. However, patients with stage 3 macular pucker that had hyporeflective intraretinal cystoid spaces between the outer nuclear and outer plexiform layers, and in the inner nuclear layer were also excluded from the present study.

### 2.3 Cumulative dissipated energy (CDE)

The CDE values in torsional and longitudinal phacoemulsification modes were automatically calculated by the device and displayed on the monitor of the phacoemulsification system.

### 2.4 Corneal endothelial cell density (ECD)

The measurement of corneal ECD was conducted using specular microscopy (TOPCON SP-3000P) pre-operatively and post operatively. To ensure consistency in ECD evaluations, the same equipment was utilized for all measurements. To maintain accuracy and reduce variability the cell counting was carried out automatically via the device.

### 2.5 Macular thickness

Pre- and post-operative macular thickness was measured using ocular coherence tomography (Zeiss Cirrus 5,000 HD-OCT). All measurements were taken with the same equipment to ensure consistency for macular thickness evaluations.

### 2.6 Statistical analysis

**Figure 2:**
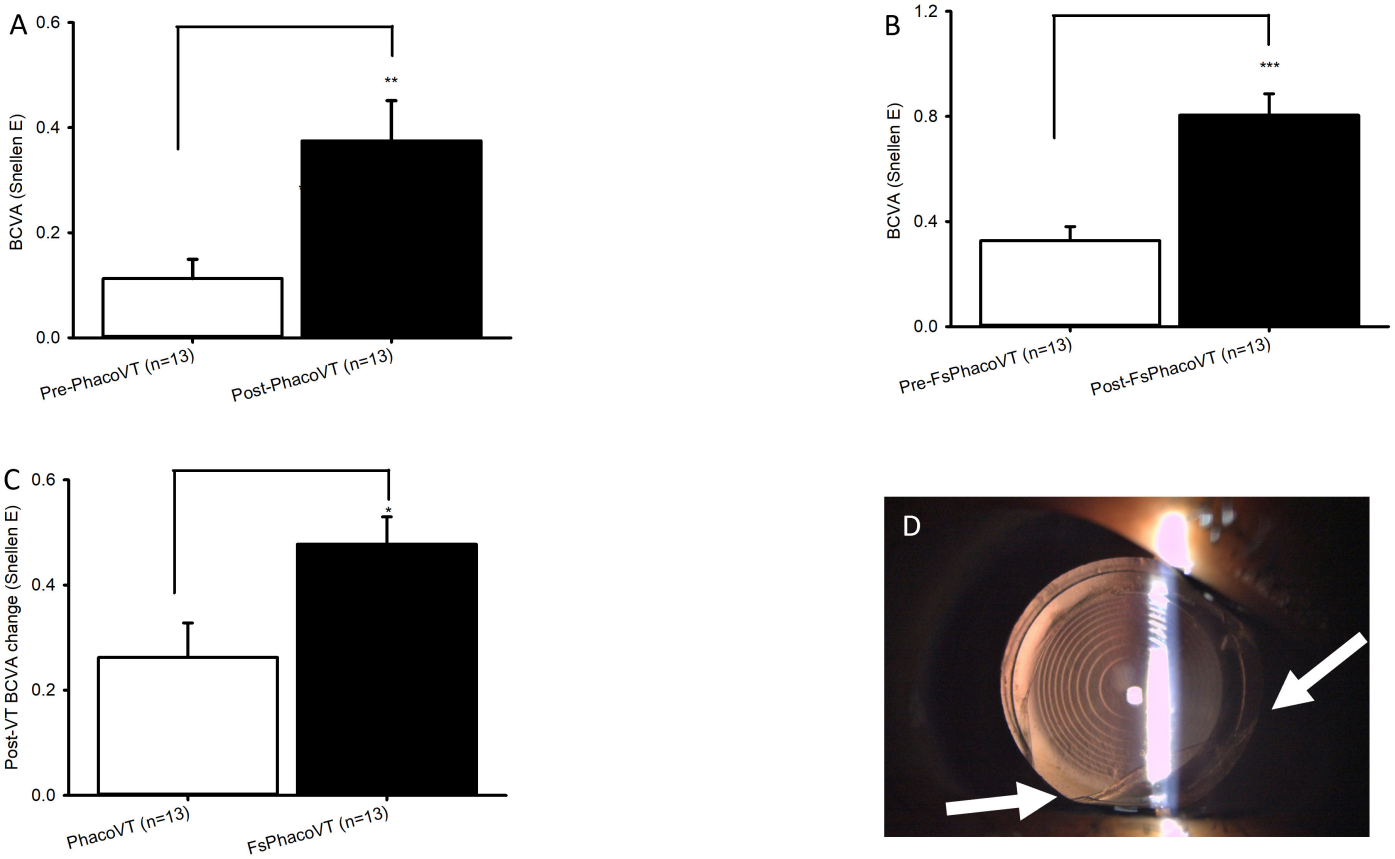
BCVA in Snellen and complications induced during femtosecond laser. The impaired pre-operative BCVAs and significantly (***, *P* < 0.001/**, *P* < 0.01; **A/B**) improved post-operative BCVAs were demonstrated in Group 1 **(A)** versus Group 2 **(B)**. Moreover, the pre- and post-operative BCVA alteration between Group 1 and Group 2 was significant (*, *P* < 0.05; **C**). As for Group 2, one eye had suction cup loss at 3 seconds of CCC due to the patient's small eye, which consequently led to anterior capsule tear at 7 O'C despite usage of a relaxing lateral canthotomy **(D)**. FsPhacoVT, femtosecond laser assisted phacovitrectomy+pars plana vitrectomy; PhacoVT, phacovitrectomy+pars planavitrectomy; BCVA, Best corrected visual acuity; CCC, continuous curvilinear capsulorhexis.

**Figure 3:**
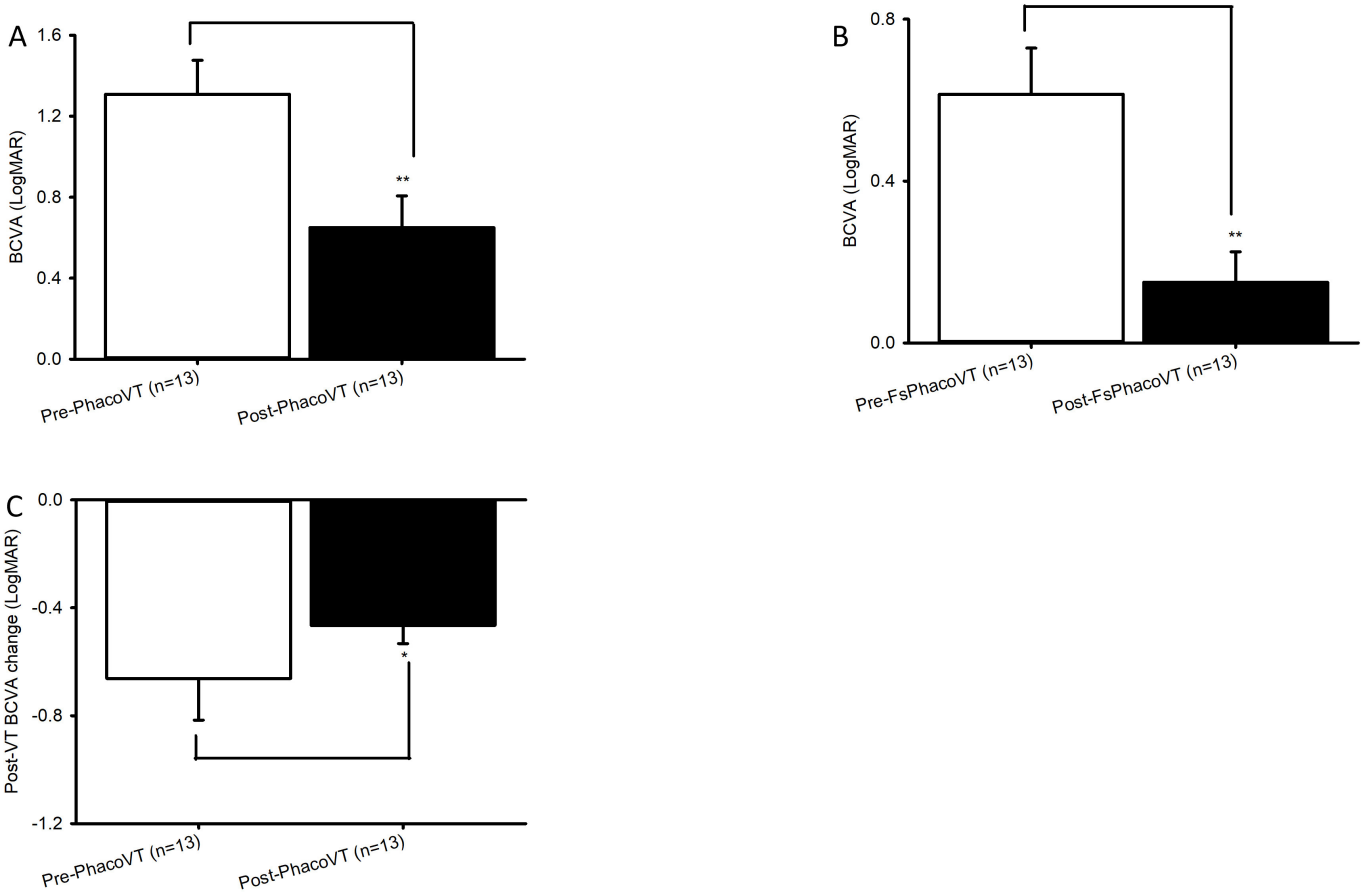
BCVA in LogMAR. The impaired pre-operative BCVAs and significantly (**, *P* < 0.01/**, *P* < 0.01; **A/B**) improved post-operative BCVAs were revealed in Group 1 **(A)** versus Group 2 **(B)**. Moreover, the pre- and post-operative BCVA alteration between Group 1 and Group 2 was significant (*, *P* < 0.05; **C**). FsPhacoVT, femtosecond laser assisted phacovitrectomy+pars plana vitrectomy; PhacoVT, phacovitrectomy+pars planavitrectomy; BCVA, Best corrected visual acuity.

**Figure 4:**
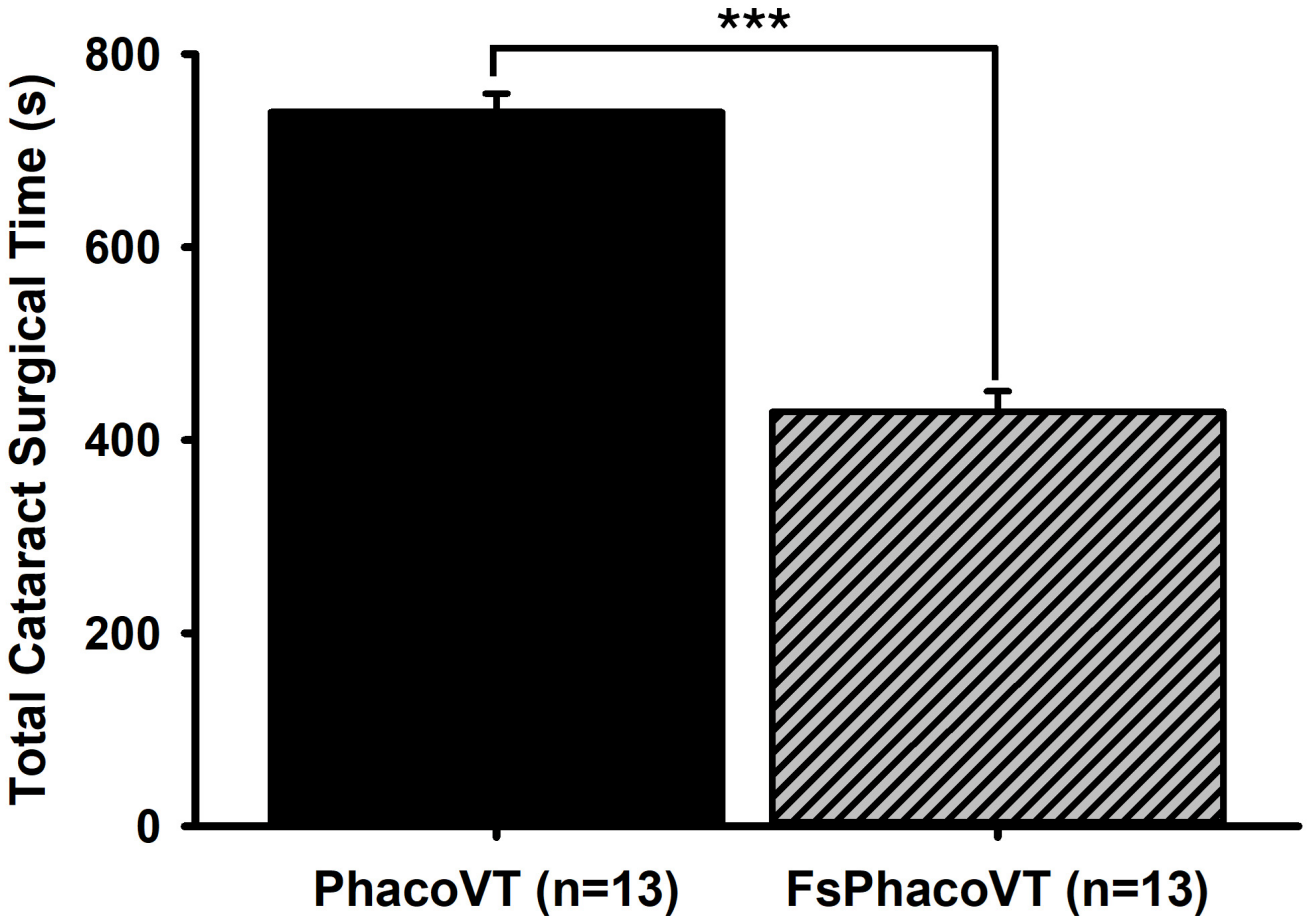
Phacoemulsification cataract surgical time. Femtosecond laser assistance very significantly (***, *P* < 0.001) shortened the cataract surgical time (s) in phacovitrectomy in contrast to that in traditional phacovitrectomy. FsPhacoVT, femtosecond laser assisted phacoemulsification+IOL implantation+pars plana vitrectomy; PhacoVT, phacoemulsification+IOL implantation+pars plana vitrectomy; BCVA, Best corrected visual acuity.

**Figure 5:**
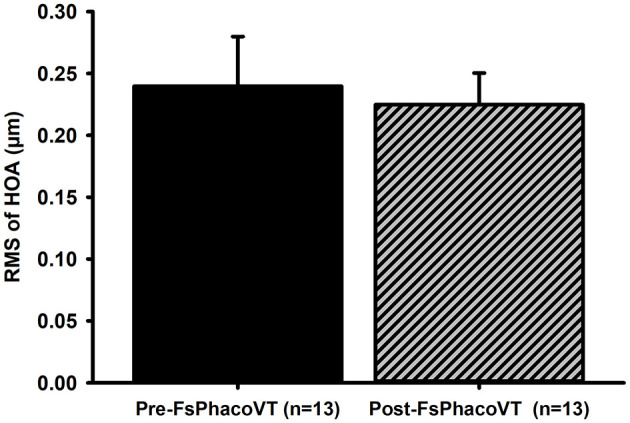
High order aberrations. The cornea wavefront data indicated that femtosecond laser did not significantly alter high order aberrations (HOAs) postoperatively (from 0.24 to 0.22 μm) in Group 2. FsPhacoVT, femtosecond laser assisted phacoemulsification+IOL implantation+pars plana vitrectomy; PhacoVT, phacoemulsification+IOL implantation+pars plana vitrectomy; RMS, root mean square; HOA, high order abberations.

The data used for statistical analysis included BCVAs in Snellen E/logMAR, cataract surgical time and total RMS (HOAs) of FSL-assisted/traditional phacoemulsification. Graphs in Figures 2–5 were drawn utilizing Sigma Plot 12.5 (Jandel Scientific, Corte Madera, CA) and statistical analysis was conducted using SPSS for Windows version 23.0.

All data were expressed as mean ± standard error. An unpaired Student's *t*-test was used to compare two independent groups. A probability value of *P* < 0.05 was considered significant.

## 3 Results

### 3.1 Demographics

Based on the inclusion/exclusion criteria, there were 26 eyes (*n* = 15 = 57.69% eyes of females) in 24 patients of stage 3 macular pucker and medium density nuclear cataract, with a mean age of 68.19 ± 2.03 years. Two age-matched groups (*P* = 0.12) were identified: phacovitrectomy without femtosecond laser assistance (PhacoVT; Group 1; *n* = 13; 71.38 ± 3.00 years old), or phacovitrectomy with FSLA (FsPhacoVT; Group 2; *n* = 13; 65.00 ± 2.60 y-o). In this case, 13 eyes received PhacoIOL-PPVT without FSLA (Group 1, Table 1) and 13 eyes underwent FSLA PhacoIOL-PPVT (Group 2, Table 2). The mean follow-up period was 36.04 ± 3.42 months.

**Table 1:** Features of candidates who all had cataract received phacovitrectomy (*n* = 13 eyes).

**No**.	**Sex/age/eye Dx**	**Peel**	**Preop BCVA, E (LogMAR)**		**Postop BCVA,E (LogMAR)**	**F-U, months**	**Complications**
i1	F/L	Pucker	+	0.05 (1.3)	0.1 (1.0)	21	-
i2	M/L	Pucker	+	0.03 (1.5)	0.6 (0.2)	21	-
i3	F/L	Pucker	+	0.3 (0.5)	0.8 (0.1)	17	-^*^Nu drop
i4	F/L	Pucker	+	0.1 (1)	0.6 (0.2)	16	-
i5	F/R	Pucker	+	0.3 (0.5)	0.63 (0.2)	17	-
i6	M/R	Pucker	+	0.014 (1.9)	0.03 (1.5)	26	-
i7	M/R	Pucker	+	0.014 (1.9)	0.014 (1.9)	32	-
i8	F/L	Pucker	+	0.014 (1.9)	0.6 (0.2)	30	-
i9	F/R	Pucker	+	0.014 (1.9)	0.1 (1)	29	-
i10	M/L	Pucker	+	0.014 (1.9)	0.5 (0.3)	33	-
i11	M/R	Pucker	+	0.014 (1.9)	0.1 (1.0)	39	-
i12	F/L	Pucker	+	0.3 (0.5)	0.5 (0.3)	30	-
i13	M/L	Pucker	+	0.3 (0.5)	0.3 (0.5)	20	-

phacovitrectomy, phacoemulsification+posterior chamber intraocular lens (IOL) implantation+pars plana vitrectomy; Dx, diagnosis; Peel, epiretinal/internal limiting membrane peeling; Preop/Postop, preoperative/postoperative; BCVA, best corrected visual acuity; E, Snellen E; LogMAR, logarithm of the minimum angle of resolution; M, Male; F, Female; R, Right; L, Left; Pucker, Macular pucker; ^*^dislocated nucleus owing to posterior capsule rupture.

**Table 2:** Features of candidates who all had cataract and received femtosecond phacovitrectomy (*n* = 13 eyes).

**No**.	**Sex/age/eye Dx**	**Peel**	**Preop BCVA, E (LogMAR)**		**Postop BCVA,E (LogMAR)**	**F-U, months**	**Complications**
ii1	F/R	Pucker	+	0.3 (0.5)	0.8 (0.1)	80	-
ii2	F/L	Pucker	+	0.2 (0.7)	0.9 (0.05)	69	-
ii3	F/R	Pucker	+	0.3 (0.5)	1.0 (0)	63	-
ii4	F/L	Pucker	+	0.05 (1.3)	0.2 (0.7)	57	^*^cup loss & ant cap tear
ii5	F/L	Pucker	+	0.4 (0.4)	1.0 (0)	56	-
ii6	M/L	Pucker	+	0.05 (1.3)	0.7 (0.2)	56	-
ii7	M/R	Pucker	+	0.5 (0.3)	1.0 (0)	47	-
ii8	F/L	Pucker	+	0.4 (0.4)	0.9 (0.05)	37	-
ii9	F/R	Pucker	+	0.4 (0.4)	0.8 (0.1)	34	-
ii10	M/L	Pucker	+	0.6 (0.2)	1.0 (0)	33	-
ii11	M/R	Pucker	+	0.6 (0.2)	1.0 (0)	35	-
ii12	M/R	Pucker	+	0.4 (0.4)	1.0 (0)	25	-
ii13	F/L	Pucker	+	0.05 (1.3)	0.16 (0.8)	22	-

phacovitrectomy, phacoemulsification+posterior chamber intraocular lens (IOL) implantation+pars plana vitrectomy; Dx, diagnosis; Peel, epiretinal/internal limiting membrane peeling; Preop/Postop, preoperative/postoperative; BCVA, best corrected visual acuity; E, Snellen E; LogMAR, logarithm of the minimum angle of resolution; M, Male; F, Female; R, Right; L, Left; Pucker, Macular pucker; ^*^cup loss and anterior capsule tear.

### 3.2 BCVA

Results indicated that BCVA improved in 24 eyes (92.3%; Tables 1, 2), although two eyes in Group 1 showed no change in BCVA. Pre-operatively, the BCVAs in both groups showed no statistically significant difference (*P* > 0.05). The initially impaired pre-operative BCVA significantly improved post-operatively, evidently in Group 1 (0.11 ± 0.04 Snellen E/1.31 ± 0.17 LogMAR to 0.38 ± 0.08 Snellen E/0.65 ± 0.16 LogMAR; *P* < 0.01/*P* < 0.01; Figures 2A, 3A), and even more pronounced in Group 2 (0.33 ± 0.05 Snellen E/0.60 ± 0.11 LogMAR to 0.80 ± 0.08 Snellen E/0.15 ± 0.08 LogMAR; *P* < 0.001/*P* < 0.01; Figures 2B, 3B). The pre- and post-operative improvements in BCVA were statistically different (*P* < 0.05) when comparing Group 1 with Group 2. In Group 1 the improvement was 0.26 ± 0.07 Snellen E (−0.66 ± 0.15 logMAR) whereas in Group 2 it was 0.48 ± 0.05 Snellen E (−0.46 ± 0.07 LogMAR).

Furthermore, within the subgroup of patients with macular pucker, the results demonstrated 1 out of 13 (7.7%) eyes (in Group 1) and 11 out of 13 (84.6%) eyes (in Group 2) achieved a postoperative BCVA of ≥0.7 Snellen E or ≤ 0.2 LogMAR (Tables 3, 4). As mentioned, within this subgroup of patients, pre- and post-operative BCVA change importantly expressed a significant difference (*P* = 0.02) between Groups 1 and 2, with improvements of 0.26 ± 0.07 Snellen E (Group 1; *n* = 13) vs. 0.48 ± 0.05 Snellen E (Group 2; *n* = 13). In terms of complications, there were two intraoperative sequelae, one within each of Group 1 and 2, with the latter group experiencing less serious complications. In Group 1, one eye had a dislocated nucleus owing to an anterior capsule tear at 10 O'C, extending to a posterior capsule rupture. This serious complication necessitated an IOL implantation in the ciliary sulcus. As for Group 2, one eye experienced suction cup loss at 3 s during CCC due to the patient's small palpebral fissure, which consequently led to anterior capsule tear at 7 O'C despite use of a relaxing lateral canthotomy (Figure 2D). Importantly, no patients in Group 2 experienced intraocular lens subluxation.

**Table 3:** Preoperative clinical characteristics of patients without versus with femtosecond assisted phacoemulsification followed by vitrectomy.

**Parameter**	**PhacoVT (Group 1)**	**FsPhacoVT (Group 2)**
No. of eyes (*n*)	*n* = 13	*n* = 13
Eyes of ♂/♀ (♀ %)	6/7 (54%)	5/8 (62%)
Od/Os eye (Od %)	5/8 (39%)	5/8 (39%)
MP/Op age (year; *n*)	71.38 ± 3.00 (*n* = 13)	65.00 ± 2.60 (*n* = 13)
MP/ Pre-op BCVA E (LogMAR)	0.11 ± 0.04 (1.31 ± 0.17; *n* = 13)	0.33 ± 0.05 (0.61 ± 0.11; *n* = 13)

The values were presented as mean ± standard error of the number of eyes. FsPhacoVT, femtosecond laser assisted phacoemulsification+IOL implantation+pars plana vitrectomy; PhacoVT, phacoemulsification+IOL implantation+pars plana vitrectomy; BCVA, best corrected visual acuity; E, Snellen E; LogMAR, logarithm of the minimum angle of resolution; Od, right; Os, left; MP, macular pucker.

**Table 4:** Postoperative outcomes of patients without versus with femtosecond assisted phacoemulsification followed by vitrectomy.

**Parameter**	**PhacoVT (Group 1)**	**FsPhacoVT (Group 2)**
No. of eyes (n)	*n* = 13	*n* = 13
follow up (months)	25.46 ± 2.02	33.33 ± 4.87
MP/Post-op BCVA E (LogMAR)	0.38 ± 0.08 (0.65 ± 0.16; *n* = 13)	0.8 ± 0.08 (0.15 ± 0.07; *n* = 13)
MP/Post-op BCVA ≥0.7 E (0.2 LogMAR)	7.7% (=1/13)	84.6% (=11/13)
MP/Post-op BCVA (LogMAR) change	0.26 ± 0.07 (−0.66 ± 0.15; *n* = 13)	0.48 ± 0.05^*^ (−0.46 ± 0.07; *n* = 13)
**Complications**		
Posterior capsule tear	*n* = 1†	*n* = 0
Anterior capsule tear	*n* = 0	*n* = 1††

The values were presented as mean ± standard error of the number of eyes. The unpaired Student's t-test was used when comparing two independent groups. A probability value of P <0.01/0.05 was considered ^**^very significant/^*^significant difference (Group 1 vs. Group 2). FsPhacoVT, femtosecond laser assisted phacoemulsification+IOL implantation+pars plana vitrectomy; PhacoVT, phacoemulsification+IOL implantation+pars plana vitrectomy; BCVA, best corrected visual acuity; E, Snellen E; LogMAR, logarithm of the minimum angle of resolution; MP, macular pucker; VH, vitreous hemorrhage; MH, macular hole; RD, retinal detachment. † dislocated nucleus owing to posterior capsule rupture; ††suction cup loss at 3 s due to the patient's small eye despite a relaxing canthotomy with consequent anterior capsule tear at 7 O'C.

### 3.3 Surgical time and HOA

The total cataract surgical time (Figure 4) showed a highly significant difference (*P* < 0.001) between the two groups. In Group 1 (PhacoVT; *n* = 13) the mean total cataract surgical time was 740.00 ± 19.12 s. In contrast, Group 2 (FsPhacoVT; *n* = 13) demonstrated a markedly shorter mean surgical time of 429.46 ± 21.41. Additionally, vitrectomy time was also assessed, revealing a mean time of 69.8 ± 47.09 min (*n* = 13) compared to 81.40 ± 7.10 min (*n* = 13) Group 1 (PhacoVT). A trend toward a shorter vitrectomy time with FsPhacoVT was observed although this difference was not significant (*P* > 0.05).

In Group 2, a comparison of preoperative and postoperative corneal wavefront higher order aberrations (HOAs) showed no significant difference (*P* = 0.9). The mean preoperative HOAs were 0.24 ± 0.04, and postoperative HOAs were 0.22 ± 0.03.

### 3.4 Cumulative dissipated energy

**Figure 6:**
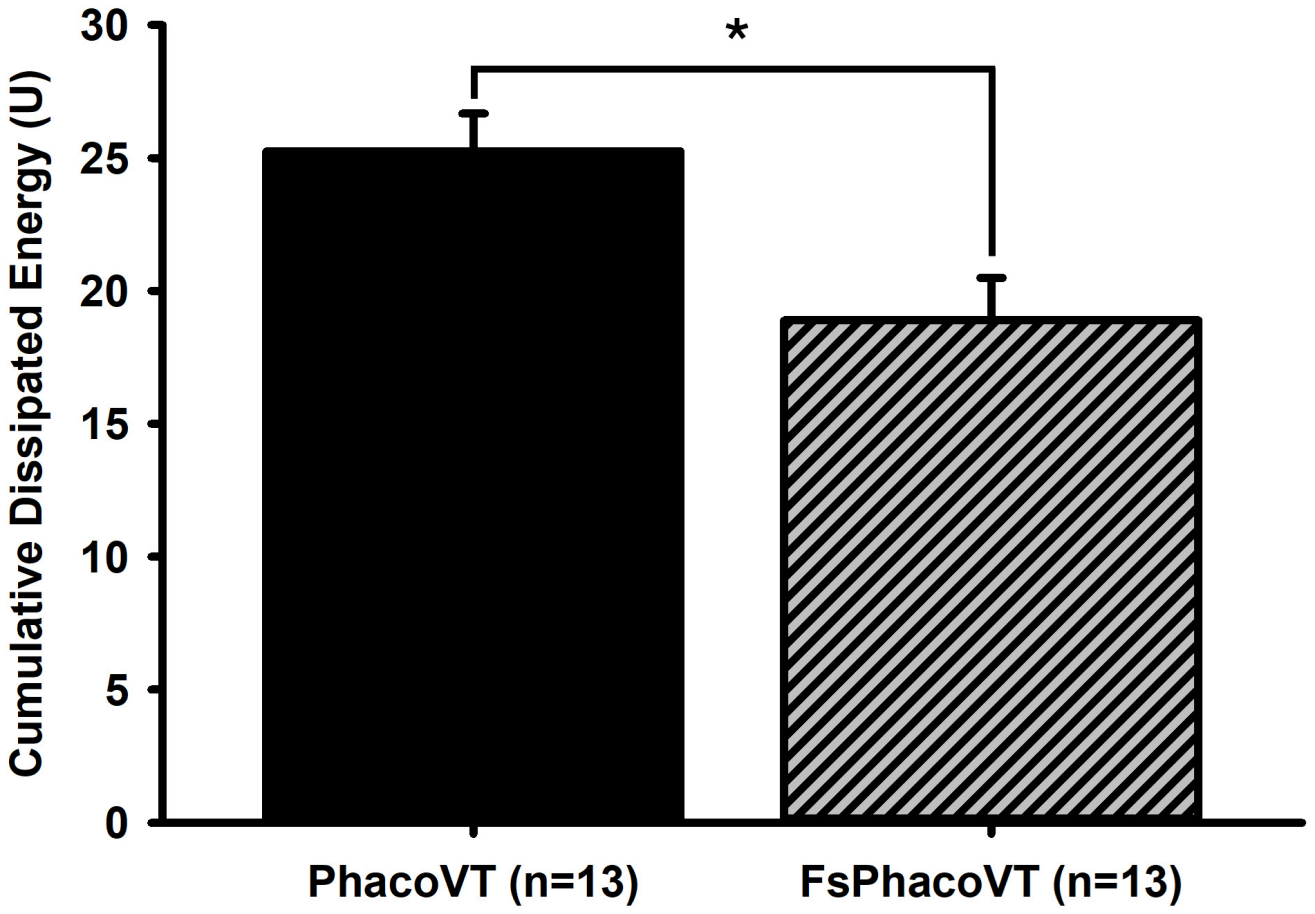
Cumulative dissipated energy (CDE). The CDE values were significant (*, *P* < 0.01) lower in Group 2 (18.90 ± 1.59; FsPhacoVT; *n* = 13) than in Group 1 (25.24 ± 1.42; PhacoVT; *n* = 13). FsPhacoVT, femtosecond laser assisted phacoemulsification+IOL implantation+pars plana vitrectomy; PhacoVT, phacoemulsification+IOL implantation+pars plana vitrectomy; CDE, cumulative dissipated energy.

The mean CDE values were analyzed for both groups (Figure 6). In Group 1 (PhacoVT; *n* = 13), the mean CDE was 25.24 ± 1.42. In Group 2 (FsPhacoVT; *n* = 13) the mean CDE was 18.90 ± 1.59. The difference between the two groups were statistically significant (*p* < 0.01), with Group 2 exhibiting a 25.15% decrease in CDE value compared to Group 1.

### 3.5 Endothelial cell density

**Figure 7:**
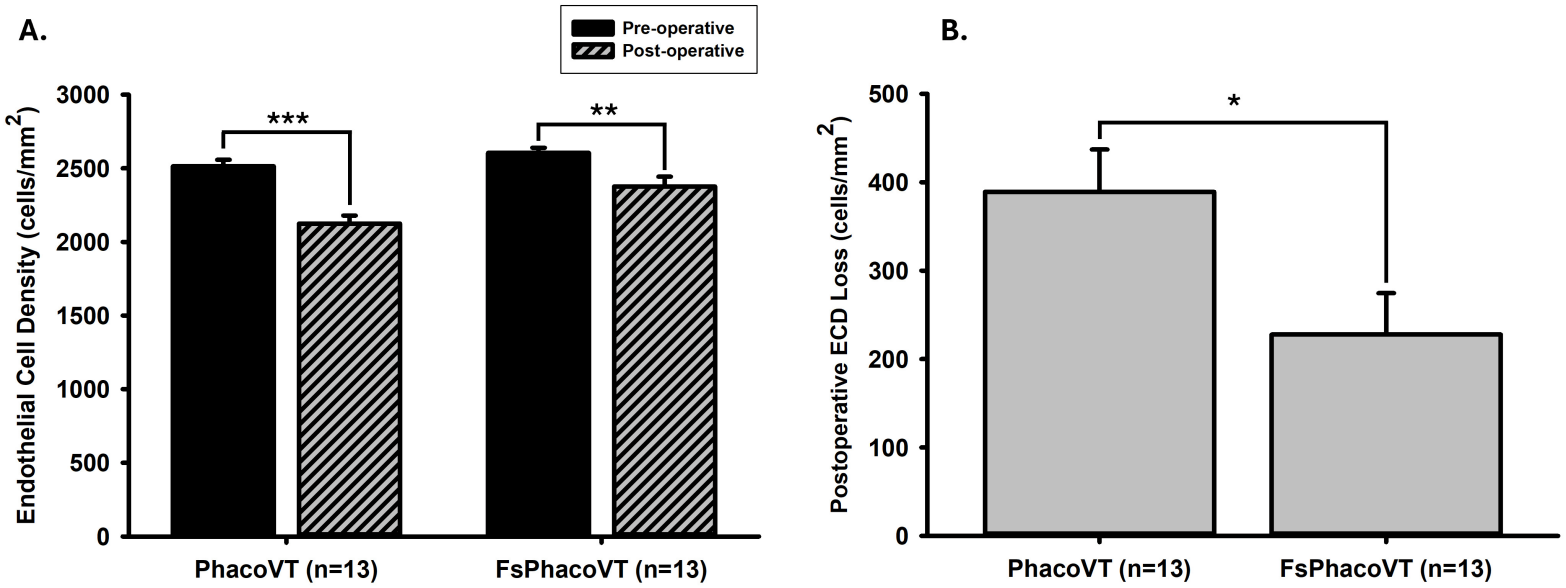
Endothelium cell density (ECD). **(A)** Prior to the FSLA or conventional phacovitrectomy, the ECD was recorded as 2,605.00 ± 34.34 or 2,514.13 ± 43.88 cells/mm^2^ (*n* = 13). Following the operation, a significant (**, *P* < 0.01 or ***, *P* < 0.001) decrease in ECD was observed, with a post-operative value of 2,377.24 ± 65.96 or 2,124.98 ± 54.21 cells/mm^2^ (*n* = 13). There was no significant difference (*P* = 0.120) in the pre-operative ECD between two groups. **(B)** Notably, there was a significant post-operative ECD loss (389.15 ± 47.87 cells/mm^2^; *, *P* < 0.05) following conventional phacovitrectomy than the one (227.77 ± 46.85 cells/mm^2^) after FSLA phacovitrectomy. FsPhacoVT, femtosecond laser assisted phacoemulsification+IOL implantation+pars plana vitrectomy; PhacoVT, phacoemulsification+IOL implantation+pars plana vitrectomy; FSLA, femtosecond laser assissted; ECD, endothelial cell density.

In Figure 7A, prior to the FSLA or conventional phacovitrectomy, the ECD was recorded at an average of 2,605.00 ± 34.34 or 2,514.13 ± 43.88 cells/mm^2^ (*n* = 13). Following the operation, a significant (*P* < 0.01 or 0.001) decrease in ECD was observed, with a post-operative average of 2,377.24 ± 65.96 or 2,124.98 ± 54.21 cells/mm^2^ (*n* = 13). In terms of the baseline, there was no significant difference (*P* = 0.120) in the pre-operative ECD between the two groups. Of note, there was a significant post-operative ECD loss (389.15 ± 47.87 cells/mm^2^; *P* < 0.05) following conventional phacovitrectomy than the one (227.77 ± 46.85 cells/mm^2^) after FSLA phacovitrectomy (Figure 7B).

### 3.6 Macular thickness

**Figure 8:**
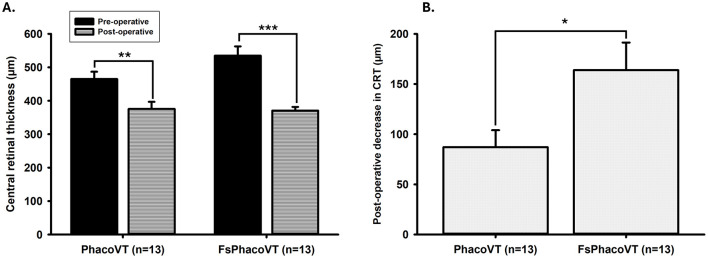
Central retinal thickness (CRT). **(A)** Pre-operative and post-operative CRT values in Group 1 and Group 2. In Group 1, the mean pre-operative CRT was 465.08 ± 21.68 μm (*n* = 13), which decreased significantly to 375.77 ± 21.18 μm post-operatively (**, *P* < 0.01). In Group 2, the mean pre-operative CRT was 534.69 ± 27.89 μm (*n* = 13), which decreased significantly to 370.77 ± 10.84 μm post-operatively (***, *P* < 0.001). No significant difference was observed in pre-operative CRT between the two groups (*P* > 0.05). **(B)** Change in CRT from pre-operative to post-operative values. The reduction in CRT was 87.08 ± 16.94 μm in Group 1 and 163.92 ± 27.50 μm in Group 2, with the reduction in Group 2 being significantly greater (*, *P* < 0.05). FsPhacoVT, femtosecond laser assisted phacoemulsification+IOL implantation+pars plana vitrectomy; PhacoVT, phacoemulsification+IOL implantation+pars plana vitrectomy; CRT, central retinal thickness.

In Group 1 (PhacoVT), the mean pre-operative central retinal thickness (CRT) was 465.08 ± 21.68 μm (*n* = 13). In Group 2 (FsPhacoVT), the mean pre-operative CRT was 534.69 ± 27.89 μm (*n* = 13). There was no significant difference in pre-operative CRT between the two groups (*P* > 0.05; Figure 8A). Post-operatively, the mean CRT in Group 1 decreased to 375.77 ± 21.18 μm (*P* < 0.01), while in Group 2, it decreased to 370.77 ± 10.84 μm (*P* < 0.001; Figure 8A). The reduction in CRT was 87.08 ± 16.94 μm in Group 1 and 163.92 ± 27.50 μm in Group 2, with the reduction in Group 2 being significantly greater (*P* < 0.05; Figure 8B).

### 3.7 Palpebral fissure length, canthotomy, synechiotomy or small/eccentric pupil

In this study, there was one case with suction cup loss at 3 s and anterior capsule tear due to the female patient's smaller (21.5 mm) than average (Chinese female: 29.4 mm) palpebral fissure length (17), representing a calculated difference of 26.9%. This resulted in the FSL procedure being performed under a considerable retrobulbar tension even after a pre-processed lateral canthotomy. Moreover, a “second” FSL procedure was restarted for her to complete the “previously incomplete” lens fragmentation. Thus, it is suggested to choose a suction cup with a smaller outer diameter.

In cases of uveitis induced posterior synechiae, healon was utilized to separate the adhesions, followed by CCC and lens fragmentation. For patients with an eccentric pupil or pupil smaller than 5 mm (e.g., a pilocarpine user), CCC diameter could be adjusted to the pupil maximized model or manually adjusted to the maximized pupil size to complete the CCC. In certain cases where the pupil is relatively small ( ≤ 3 mm), a 2^nd^ manual CCC could be considered to enlarge the anterior capsulotomy size to ~5 mm.

## 4 Discussion

FSLA phacovitrectomy has been previously reported in studies involving Caucasian populations but has rarely been studied in Oriental patients. Pioneeringly, a comparison has been made between previous studies involving Caucasian patients and our present study involving Chinese patients, using various parameters and tools. Moreover, this is the first study to compare conventional with FSLA combined phacovitrectomy in Oriental patients. Furthermore, the specific issues encountered while performing FSLA phacovitrectomy, along with methods of troubleshooting, will also be discussed in detail below.

### 4.1 FSLA phacovitrectomy: caucasian vs. chinese patients

To better understand the impact of FSLA phacovitrectomy and its application across different ethnicities, we compare the outcomes of Caucasian studies with our present results. Gomez-Resa et al. (6) published a study including 21 eyes of 21 patients who underwent 23-G PPVT and FSLA cataract surgery (6). The most common indication for surgery, similar to our study, was macular pucker. The authors reported that all the IOLs remained well positioned at the 3-month follow-up. Their results further demonstrated a pre-/post-FSLA phacovitrectomy BCVA change to be −0.69 ± 0.20 in LogMAR units. In contrast to their findings, our present study demonstrates a different visual outcome for the FSLA phacovitrectomy group (−0.46 ± 0.07 LogMAR; Table 4). This difference could possibly be attributed to both the present pre-operative BCVA differences (0.81 LogMAR vs. 0.60 LogMAR) among recruited patients and the differences in ethnicity between the study groups. Importantly, both studies have supported that the FSLA in phacovitrectomy possesses merits over traditional techniques for treating patients with macular/vitreoretinal disorders.

Similarly, Yilmaz et al. (5) investigated the safety and efficiency of combined FSL-assisted cataract surgery with sutureless 25-G and 27-G vitrectomy (5). The study included 23 patients with varying vitreoretinal pathologies (e.g., macular pucker). The authors stated that FSLA phacovitrectomy offers highly accurate capsulotomy and nucleus fragmentation even in the absence of a red fundus reflex as well as precise IOL stability during fluid-gas exchange and the postoperative period with intraocular gas tamponade (5). Disadvantages of FSLA phacovitrectomy can be subconjunctival hemorrhage, suction loss and miosis (5). Yilmaz et al. (5) suggested that to prevent chemosis, FSL can be initially conducted under topical anesthesia, followed by phacoemulsification performed under retrobulbar (6) or general anesthesia, which was similarly adopted in the present study.

Furthermore, improved visual outcomes (7) and reduced vitreous loss (18) have been reported in studies involving Caucasian populations. However, complications like posterior capsule rupture, zonular dialysis (19), and increased CDE in advanced cataracts (20) remain concerns. In Spain and Brazil, FLACS improved outcomes but was linked to miosis, corneal edema, and ocular hypertension (21).

FSL-assisted phacovitrectomy shows promise in improving surgical precision and outcomes, but variations in preoperative conditions, surgical techniques, and patient demographics must be accounted for. Differences in outcomes between Caucasian and Chinese patients, along with reported complications, emphasize the importance of tailored approaches. Further research in diverse populations will help refine these techniques and address challenges in complex cases.

### 4.2 FSL assistance in phacovitrectomy: insights from Asian populations

While recent studies have expanded our understanding of FSL-assisted vitrectomy in diverse populations, few reports have discussed the effect of FSL in combined phacovitrectomy, with most focusing on vitrectomized eyes. In Asia, studies have both reported both advantages and complications associated with FLACS in vitrectomy.

#### 4.2.1 Southeast Asia

Studies on Indian populations have demonstrated the outcomes of FSLA in phacovitrectomy. Kelkar et al. (22) reported improved visual acuity in patients with macular pucker, while Bali et al. (3) noted reduced intraoperative time, ability to maintain clear retinal view at all times, and better IOL centration with FSLA in phacovitrectomy. Additionally, Asif et al. (8) demonstrated FSLA in vitrectomized eyes reduced intraoperative ultrasound energy, endothelial cell loss, and changes in central corneal thickness (CCT) compared to conventional phacoemulsification. These findings align with our results, which showed a significant reduction in corneal endothelial cell loss and shorter surgical times. However, it is important to acknowledge anatomical differences exist between Southeast Asians (e.g., Indians) and East Asians (e.g., Chinese), such as variations in anterior chamber depth, axial length, and corneal curvature, which may influence the applicability of these findings to our study population (23–25).

#### 4.2.2 East Asia

In comparable East Asian populations, several studies have further highlighted the advantages and demonstrated the complications of FSL-assisted techniques in vitrectomy. Cai et al. (15) reported that FLACS improved surgical safety and accelerated visual rehabilitation in vitrectomized eyes with hard nuclear cataracts in Chinese patients (15). More specifically, their study demonstrated reduced cumulative dissipated energy (CDE) and better recovery of endothelial cell loss. Their findings corroborate the results of our study. Specifically, their study demonstrated reduced cumulative dissipated energy (CDE) and better recovery of endothelial cell loss, findings that align with the results of our study. However, their focus was on vitrectomized eyes undergoing delayed cataract surgery rather than combined phacovitrectomy, which limits direct comparability to our study.

Kubota et al. (26) investigated the complications of FSL-assisted cataract surgery in combined phacovitrectomy in Japanese patients (26). Although they documented rare cases of endophthalmitis following the procedure, these were attributed to surgical factors rather than the use of FSL. While their study did not directly compare FSL-assisted techniques with conventional phacovitrectomy, it demonstrated that FSLA in combined procedures is safe and provides precise capsulotomies, which reduce the risk of IOL prolapse into the anterior chamber during gas tamponade. Their findings support the use of FSL in combined procedures, emphasizing its role in improving surgical outcomes and reducing complications.

While these studies demonstrate the safety of FSLA in vitrectomy and, in some cases, its applicability to Oriental populations, there are inherent limitations to their generalizability to our study. To our knowledge, only three studies to date have provided direct comparisons between FSL-assisted and conventional phacoemulsification in vitrectomized eyes. Among these, Cai et al. (15)'s study is the only one that compares FSLA in vitrectomy with conventional phacovitrectomy in Oriental patients. However, their study focused on vitrectomized eyes, which represents an inherently different procedure with distinct risks—a point that will be further discussed in the section on complications of FSLA in vitrectomy. Additionally, these studies did not directly compare FSLA and conventional phacovitrectomy by isolating disease stages. In contrast, our study specifically compared patients with the same disease stage—stage 3 macular pucker and medium-density nuclear cataract—thereby minimizing external variables such as disease severity or condition, which could otherwise influence the outcomes and safety of FSLA versus conventional phacovitrectomy.

### 4.3 Trouble shooting for FSL assisted phacovitrectomy

#### 4.3.1 Incomplete capsulotomy

In a female with the eye that received FSL-assisted cataract surgery, the use of a face mask (worn due to COVID-19 pandemic official regulations) resulted in an incomplete (mainly nasal side) CCC flap (>180°) that was intentionally lifted with a Sinski hook while processing a successful nuclear fragmentation/cortex aspiration. In context with this, a male patient with a considerably smaller eye (excluded in the samples due to recurrent RRD) had a “360 degrees” capsule rupture from posterior to anterior (cut into 2 hemispheres) during PPVT for the treatment of fresh RRD. Two weeks later, FSL was selected to assist in creating an anterior capsulotomy as large as 8 mm. Subsequently, the FSL fragmented nucleus in the capsular bag was carefully engaged/emulsified with the phacoemulsification tip. Finally, the IOL was implanted in the ciliary sulcus before the careful and successful aspiration of the residual lens cortex with the simultaneous assistance of an intraocular forceps to hold and stabilize the IOL body.

#### 4.3.2 Nucleus fragmentation

FSL power (for Z8/LenSAR/Catalys) was schematically set at 1/7/8 μJ for medium density nuclear cataract patients. For safety reasons, manual CCC/half lens depth fragmentation could be considered in patients with shallow anterior chamber.

### 4.4 FSL assisted versus traditional phacovitrectomy

FSLA phacovitrectomy has benefits over conventional phacovitrectomy due to shorter phacoemulsification time and decreased ultrasound energy required (27). These benefits translate into decreased central corneal endothelial cell loss and reduced corneal edema, which enable better visualization during the vitreoretinal surgery (28). FSLA phacovitrectomy appears to be an efficient and safe surgical procedure providing benefits such as accurate and centralized capsulorhexis as well as pre-fragmentation of the nucleus (3, 4, 6). As mentioned previously, this is useful in maintaining the IOL within the capsular apparatus and creating less serious capsular/lenticular complications (e.g., anterior capsule tear in Group 2 vs. extensive capsule rupture with subsequent nucleus dislocation in Group 1). Overall, FSL-assisted phacovitrectomy is a safe procedure that enhances IOL stabilization and visual outcomes (17, 28).

Building on these advantages, the present study highlights several key areas where FSL-assisted phacovitrectomy outperforms conventional techniques. Most notably, significantly better visual outcomes were achieved post-operatively when comparing Group 1 (PhacoVT) and Group 2 (FsPhacoVT). While both groups exhibited significant improvement of BCVAs post-operatively, Group 2 demonstrated a statistically superior improvement in visual outcomes (Group 1 vs. Group 2: 0.26 Snellen E/−0.66 LogMAR vs. 0.48 Snellen E/−0.46 LogMAR). Notably, preoperative BCVA did not differ significantly between groups, indicating that the superior outcomes in Group 2 are likely attributable to the advantages of FSL-assisted phacovitrectomy rather than baseline differences. The positive visual outcomes described above could be attributed to several factors associated with FSL-associated phacovitrectomy. These include reduced endothelial cell count loss (28), less serious complications (29–32), and significantly shorter effective phacoemulsification time due to pre-CCC and pre-fragmentation, resulting in reduced ultrasound energy for phacoemulsifying and decreased inflammation (27). These factors collectively contribute to faster post-operative recovery, as reduced inflammation and tissue trauma allow patients to achieve functional vision sooner. This is supported by our study where significantly more patients in Group 2 achieved a postoperative BCVA of ≥ 0.7 Snellen E or ≤ 0.2 LogMAR, compared to Group 1. This suggest that FSL-assisted phacovitrectomy not only enhances visual outcomes but also improves visual rehabilitation, enabling patients to achieve functional vision within a similar follow-up period. This is further supported by Asif et al. (8), who reported a trend of earlier visual recovery in the FLACS group compared to the conventional phacovitrectomy group (8). Together, these results, highlight the potential of FSL-assisted phacovitrectomy to optimize both surgical efficiency and post-operative recovery, offering a significant advantage over conventional techniques.

In addition to visual outcomes, the reduction in surgical time is another critical advantage of FSL-assisted phacovitrectomy. When compared with traditional phacoemulsification technique, a significant reduction in total effective phacoemulsification time with FLACS is shown to be most pronounced in medium-density nuclear cataracts (31), which may provide a relevant explanation for the present results (Figure 6). The present result (Figure 4) has shown that FSLA fairly significantly shortened the cataract surgical time in phacovitrectomy (429.46 s) as compared to that in traditional phacovitrectomy (740.00 s). This reduction in total phacoemulsification time contributed to reduced corneal handling and thus decreased corneal trauma. This would also be a crucial factor in disrupting corneal endothelial integrity and increasing the likelihood of corneal odema. Additionally, in terms of vitrectomy time, although not significantly different (*P* > 0.05), had a tendency to be less (69.8 ± 47.09 min; *n* = 13) in Group 2 (FsPhacoVT) than the one (81.40 ± 7.10 min; *n* = 13) in Group 1 (PhacoVT). These findings are supported by Bali et al. (3) and Asif et al. (8), who demonstrated that the use of FSL to assist phacovitrectomy reduces total surgical time. Li et al. (33) indicated that postoperative complications of combined phacovitrectomy are largely influenced by surgical duration, increased surgical manipulation and greater inflammation associated with the combined nature of the procedure (33). As Bali et al. noted, since femtosecond laser assistance mitigates these factors, potentially reducing complications and facilitating recovering. Moreover, its benefits become even more pronounced in phacovitrectomy compared to phacoemulsification alone, given the increased surgical duration and manipulation involved (3). The shorter surgical time not only minimizes intraoperative stress on ocular tissues but also reduces post-operative inflammation, which is a key determinant of recovery speed. Kim et al. (34) indicated that longer surgical times in phacovitrectomy lead to higher rates of both intraoperative and post-operative complications. Intraoperatively, this means poorer visualization during vitrectomy, and post-operatively, it increases the risk of ERM formation and proliferative vitreoretinopathy (34). By reducing surgical time and minimizing tissue trauma, FSL-assisted phacovitrectomy offers to enhance intraoperative efficiency and improve post-operative outcomes, ultimately leading to faster recovery and better patient satisfaction.

Another significant advantage of FSL-assisted phacovitrectomy is its ability to reduce corneal endothelial trauma. FSLA phacovitrectomy also reduces corneal endothelial trauma from phacoemulsification ultrasound waves, as evidenced by analyzing the CDE values between both groups. CDE is the amount of ultrasound energy used during phacoemulsification; a lower value indicates less ultrasound energy to the eye/cornea during the procedure. Clinically, this correlates to reduced mechanical and chemical stress imposed to the eye, which is critical for preserving endothelial cell health and reducing post-operative complications (35–37). Specifically, Figure 6A shows that the CDE values were significantly (*P* < 0.01) lower in Group 2 (18.90; FsPhacoVT) than in Group 1 (25.24; PhacoVT). The reduction in time and energy required to emulsify the crystalline lens due to pre-CCC and pre-fragmentation contributes to this decrease. It is well documented that phacoemulsification ultrasound energy not only mechanically damage the corneal endothelium through its high energy physical shockwaves (35–37); but also cause chemical harm toward the endothelium through the formation of hydroxyl radicals, leading to oxidative stress and a reduction in endothelial cell count (38, 39). The significantly reduced CDE value observed in this study underscores that FSL-assisted phacovitrectomy prominently reduced ultrasound energy delivered to the cornea, thereby minimizing endothelial cell damage. Moreover, corneal wavefront data revealed that FSL did not significantly alter higher-order aberrations (HOAs) postoperatively, with values remaining stable (from 0.24 to 0.22 μm) in Group 2 (Figure 5). Reduced CDE and ultrasound energy were also evident in other studies comparing FSLA and conventional phacovitrectomy (8, 15) with Cai et al. (15)'s study demonstrating that FSLA resulted in faster reduction in corneal oedema compared to conventional phacovitrectomy. This demonstrates that FSL safely and efficiently assists in phacoemulsification and IOL implantation combined with small gauge vitrectomy, preserving corneal optical quality.

The combined effect of reduced total surgical time and phacoemulsification ultrasound energy results in decreased endothelial cell count loss. Our data regarding corneal endothelial cell counts (Figure 7) reveal pre-operative FSL-assisted phacovitrectomy at 2,605.00 cells/mm^2^ and post-operative FSL-assisted phacovitrectomy at 2,377.24 cells/mm^2^, representing a significant ECD loss of 8.7% (227.76 cells/mm^2^). In contrast, conventional phacovitrectomy resulted in a more substantial postoperative endothelial cell loss of 15.5% (389.15 cells/mm^2^; *P* < 0.001). FSLA phacovitrectomy exhibited a significantly (*P* < 0.05) smaller postoperative ECD loss compared to conventional phacovitrectomy (37). These results align with a Danish study, which previously reported a significant (*P* < 0.001) endothelial cell count loss of 19.3% in phacovitrectomy surgery performed without FSL-assistance for idiopathic ERMs (40). This preservation of endothelial cells is critical for post-operative recovery, as it minimizes the risk of corneal decompensation and chronic corneal edema which can delay visual rehabilitation. A meta-analysis and systematic review comparing FSL-assisted and conventional phacoemulsification further confirmed that FSLA significantly reduces corneal edema by lowering total ultrasound energy and preserving endothelial cell density (41). These results underscore the advantages of FSLA phacovitrectomy in minimizing endothelial cell damage and loss compared to conventional phacovitrectomy, ultimately supporting better postoperative corneal health and faster visual recovery.

The benefits of FSL-assisted phacovitrectomy extend beyond corneal preservation to improved visualization during surgery. The accurate centration of the IOL and reduced corneal damage facilitated improved visualization of the posterior chamber during the vitrectomy procedure. Kubota et al. (26) and Bali et al. (3) has indicated that FSLA resulted in improved centration of lens and allowed for a clear visualization of the fundus, likely due to reduced corneal oedema from faster surgical times and reduced handling. Kubota et al.'s study has attributed this increased visibility to a stronger capsulotomy supported by Friedman et al. (42), who indicated that the laser capsulotomies are two times stronger than manual capsulotomies. A strong and symmetrical capsulotomy reduces anterior capsule contraction and increases fundus visibility (42). This enhanced visualization may contribute to the observed differences in macular thickness between the groups (3). In Group 1, the pre-operative central retinal thickness (CRT; 465.08 ± 21.68 μm) was significantly (*P* < 0.01) reduced post-operatively (375.77 ± 21.18 μm). In contrast, Group 2 (FsPhacoVT) had a pre-operative CRT of 534.69 ± 27.89 μm which was significantly (*P* < 0.001) reduced to 370.77 ± 10.84 μm postoperatively. Despite the thicker pre-operative CRT in Group 2, there was no significant difference in pre-operative CRT between the two groups (*P* > 0.05). Notably, the reduction in post-operative CRT in Group 1 (87.08 ± 16.94 μm) was significantly (*P* < 0.05) less than that (163.92 ± 27.50 μm) in Group 2. The potentially clearer visualization in Group 2 may have allowed for a more thorough removal of the ERM/ILM, while also possibly reducing retinal injury, leading to better recovery. This may have also contributed to the greater improvement in BCVA in Group 2. While these findings suggest that FSL-assisted phacovitrectomy may be associated with a greater reduction in macular thickness, further investigation is needed to determine if this effect can be attributed primarily to FSLA or to other underlying factors.

In summary, femtosecond laser-assisted surgery significantly enhances visual outcomes in phacovitrectomy, primarily through pre-CCC and lens pre-fragmentation. The laser's ability to create a perfectly circular and centered capsulorhexis ensures optimal IOL positioning and stability, reducing the risk of IOL decentration. Additionally, the laser's capability to fragment the crystalline lens before ultrasound emulsification decreases the required ultrasound energy, minimizing the risk of corneal endothelial cell loss and subsequent inflammation. These beneficial results lead to faster postoperative visual recovery and improved corrected visual outcomes. Moreover, the integration of femtosecond lasers with vitrectomy procedures enhances visualization of the posterior segment, especially in cases where the visual pathway is obscured (e.g., cornea or vitreous), allowing for a more meticulous and precise vitrectomy. Studies have shown that patients undergoing femtosecond laser-assisted phacovitrectomy have significantly better best-corrected visual acuity postoperatively compared to those undergoing traditional methods (27). As described, this technology also reduces the incidence of complications such as posterior capsule tears and nucleus/IOL dislocation, leading to safer and more effective surgical outcomes.

### 4.5 Adverse outcomes and practical challenges of FSLA phacovitrectomy

While our study and above studies demonstrated that FSLA phacovitrectomy is a safe and effective alternative over conventional phacovitrectomy, there are still potential adverse outcomes that should be discussed with the use of FSL in phacovitrectomy.

While this study focused on epiretinal membrane (ERM) as the primary indication for vitrectomy, FSLA phacovitrectomy could present unique challenges in cases such as retinal detachment (RD) where silicone oil is required. Notably, Cai et al. observed that residual emulsified silicone oil droplets in the anterior chamber could interfere with the femtosecond laser, potentially compromising capsulotomy precision (15). This issue is particularly relevant in cases of delayed phacoemulsification in vitrectomized eyes, as an imprecise capsulotomy can hinder smooth capsular bag formation, which is critical for maintaining optimal fundus visualization (42). However, by utilizing a combined phacovitrectomy approach, as in this study, this complication was prevented by performing phacoemulsification first, thereby eliminating any residual oil before FSL application. This suggests that integrating FSL in a single-setting phacovitrectomy may provide a more effective and complication-free method for managing combined cataract and vitreoretinal cases.

Another potential disadvantage of FSLA is the risk of suction loss during the procedure, which is often related to anatomical factors such as palpebral fissure size. Gómez-Resa et al. (6) highlighted a case of suction loss during phacovitrectomy, attributing it to the requirement of an adequate palpebral fissure for proper placement of the laser cone. This is further supported by Teshigawara et al. (43), who identified a narrow lid margin as a risk factor for suction loss (43). Asano-Kato (44) conducted a study on palpebral fissure size and suction loss rates in FSL-assisted procedures, noting that Asian populations, who often have narrower palpebral fissures, are at a higher risk of this complication (44). In our study, one case of suction loss occurred in a female patient with a palpebral fissure length of 21.5 mm, significantly smaller than the average of 29.4 mm for Chinese females (17). Moreover, despite performing a lateral canthotomy, the procedure was still carried out under considerable retrobulbar tension, highlighting the challenges of FSLA in patients with narrower palpebral fissures. This suggests that Oriental patients may be at greater risk of complications such as suction loss during FSLA phacovitrectomy. However, the use of a smaller suction cup in patients with narrower palpebral fissures could help mitigate this risk.

FSLA phacovitrectomy also carries inherent risks associated with the femtosecond laser technique itself. These include complications such as capsule tags and bridges, anterior capsule tears, miosis, endothelial damage, and the aforementioned suction cup loss (31). However, these complications are relatively rare, with reported rates of 2% for suction loss, 4% for anterior capsule tears, and 3% for endothelial damage. However, these complications are relatively rare, with reported rates of 2% for suction loss, 4% for anterior capsule tears, and 3% for endothelial damage. Additionally, as FSL is a relatively new technology, there is a learning curve even for experienced surgeons. Inexperienced surgeons are at greater risk of complications such as anterior capsular tags, which can precipitate anterior capsule tears due to improper placement of the suction cup (31). They may also experience higher rates of suction loss and thermal damage to surrounding ocular structures due to suboptimal laser energy settings. However, complication rates drop significantly as surgeons become more familiar with the system, emphasizing the importance of experience and training in minimizing adverse outcomes (45). To mitigate these risks, it is advisable to have surgeons who are well-versed in FSL technology perform the procedure.

Beyond these technical challenges, the adoption of FSLA in routine practice must also consider practical factors such as cost and accessibility. The high initial investment and maintenance costs of femtosecond laser systems may limit their availability, particularly in resource-limited settings (46). Furthermore, in many public healthcare systems, FSLA procedures are often not fully covered, requiring patients to opt for private care (47). Additionally, the need for specialized training and variability in healthcare infrastructure across regions could further restrict its widespread use. Despite these barriers, the potential for reduced surgical time, fewer complications, and improved outcomes may justify the investment in settings where resources permit. Further efforts to reduce costs, improve accessibility, and provide training could help bridge the gap, making FSLA a more accessible option in diverse healthcare environments (48).

While FSLA phacovitrectomy offers significant advantages over conventional techniques, it is not without its challenges. Complications such as suction loss, anterior capsule tears, and endothelial damage, though rare, highlight the importance of careful patient selection, surgical planning, and surgeon expertise. By addressing these potential adverse outcomes, FSLA can continue to evolve as a safe and effective option for combined cataract and vitreoretinal surgery. Despite these challenges, FSLA phacovitrectomy remains a safe and viable alternative to conventional phacovitrectomy, particularly when performed by experienced surgeons and with appropriate patient selection.

### 4.6 Limitations and future research

While this study provides valuable insights into the safety and efficacy of FSL-assisted phacovitrectomy compared to conventional techniques, there are some limitations that need to be addressed. The small sample size may limit the generalizability of our findings. However, the study design was intentionally focused on a specific patient population—Oriental patients with stage 3 macular pucker and medium-density nuclear cataract—to minimize external variables and ensure internal validity. Stage 3 macular pucker represents an optimal balance where surgical intervention is both necessary and likely to yield meaningful visual improvement, while medium-density nuclear cataract provides adequate visualization for preoperative and postoperative evaluations. Although the numbers included in this study are small, this is the first to demonstrate the benefits of FSL-assisted phacovitrectomy in Oriental populations undergoing combined phacovitrectomy, while accounting for specific disease stages (stage 3 macular pucker and medium-density nuclear cataract). This focus on a well-defined patient population ensures that our findings are internally valid and provides a foundation for future research.

As a retrospective study, this investigation relies on previously collected data, which may introduction selection bias and limit the ability to control for all confounding factors. However, the use of strict inclusion criteria and standardized surgical protocols helped mitigate these limitations and ensured consistency in the analysis. Despite these constraints, the study offers meaningful insights into the advantages of FSL-assisted phacovitrectomy in this specific patient population, particularly in terms of surgical precision and postoperative outcomes.

Moving forward, larger, multicenter prospective studies are needed to validate our results and enhance their generalizability. It is recommended to conduct a comparative study with a larger sample size and matched baseline characteristics for both groups. Additionally, incorporating more comprehensive data, such as corneal endothelial cell morphology, would provide further insights and strengthen the investigation. This study underscores the potential of FSL-assisted phacovitrectomy in improving surgical outcomes, setting the stage for future research to validate and expand on these findings (3, 28, 42).

## 5 Conclusion

In this study, the assistance of femtosecond laser in phacovitrectomy emerged as a faster, safer and more efficient procedure over traditional procedures for managing cases with macular/vitreoretinal diseases. Specifically, patients who received femtosecond laser assistance demonstrated significantly improved visual outcomes, reduced complications, and shorter surgical times. The efficiency of FSL-assistance was underscored by a notable reduction in overall surgical time, ultrasound energy and corneal endothelial cell loss. FSLA also contributed to a reduced postoperative macular thickness, while maintaining stable higher order aberrations. These findings highlight the potential of FSL-assisted phacovitrectomy in enhancing surgical efficiency and patient outcomes. Further studies with larger cohorts will be essential to further validate and confirm the clinical applications of FSL-assisted phacovitrectomy.

## Author's note

This study was accepted for presentation at the 38th Asia-Pacific Academy of Ophthalmology (APAO) Congress, held from 23–26 February 2023 at the Kuala Lumpur Convention Centre (KLCC), Malaysia.

## Data Availability

The raw data supporting the conclusions of this article will be made available by the authors, without undue reservation.
